# Three-dimensional laparoscopic sleeve gastrectomy versus gastric bypass in treating obesity complicated with type 2 diabetes mellitus

**DOI:** 10.4314/ahs.v25i2.25

**Published:** 2025-06

**Authors:** Jingfeng Gu, Guiqi Wang, Feng Feng, Jian Zhang, Dongyang Xing

**Affiliations:** Department of General Surgery, the First Hospital of Hebei Medical University, Shijiazhuang, China

**Keywords:** Laparoscopic sleeve gastrectomy, gastric bypass, obesity, type 2 diabetes mellitus, efficacy

## Abstract

**Background:**

To compare and analyze the clinical efficacy and safety of Three-dimensional (3D) laparoscopic sleeve gastrectomy (LSG) and laparoscopic Roux-en-Y gastric bypass (LRYGB) in the treatment of obesity complicated with type 2 diabetes mellitus (T2DM).

**Methodology:**

A total of 132 T2DM patients were divided into LSG group (n=66) and LRYGB group (n=66). The operation time, time of postoperative gastrointestinal function recovery, postoperative hospital stay, and incidence rate of complications were compared. The glucolipid metabolism indexes [fasting plasma glucose (FPG), fasting insulin (FINS), fasting C-peptide (FCP), glycated hemoglobin A1c (HbA1c)], body weight and nutritional status [body mass index (BMI), hemoglobin (Hb)], insulin function and insulin resistance were recorded. Short-from 36 (SF-36) questionnaire was also conducted.

**Results:**

LSG group had shorter operation time, less blood loss, and quicker postoperative recovery than LRYGB group. Both groups showed significant improvements in BMI, FBG, FINS, FCP, HbA1c, and HOMA-IR. After 12 months, LRYGB group had significantly higher scores in physical function, general health, vitality, role emotional, and mental health in SF-36 questionnaire compared to LSG group. Compared with LRYGB group, LSG group had significantly decreased operation time, reduced intraoperative blood loss, and shortened postoperative off-bed time. BMI, FBG, FINS, FCP, HbA1c and HOMA-IR all markedly declined in the two groups after operation. At 12 months after operation, the scores of physical function, general health, vitality, role emotional and mental health in the SF-36 questionnaire were all remarkably higher in LRYGB group than those in LSG group.

**Conclusion:**

LSG can be used to treat obesity complicated with T2DM, which is characterized by simple operation, small trauma and quick postoperative recovery

## Introduction

Type 2 diabetes mellitus (T2DM) is a chronic disease affecting more than 400 million people around the world, and its incidence shows an increasing trend (it is estimated that the number of DM patients will reach 642 million in 2040)^1^-^3^.

Metabolic surgery can alter the secretion of gastrointestinal hormones and affect the progression of T2DM^4^. Laparoscopic Roux-en-Y gastric bypass (LRYGB) is a standard surgery for obesity in European and American countries, which has a definite and long-lasting weight-reducing effect, and can effectively stimulate the “entero-insular axis” to increase insulin secretion. At the same time, the patient's gastric volume is greatly reduced by the surgery, so that the postoperative food intake is reduced, thereby alleviating T2DM^5^,^6^. Laparoscopic sleeve gastrectomy (LSG) first reported by Hofso et al^7^ was originally used as the first-stage surgery for extremely and severely obese patients, but it was found during follow-up that 60% of patients can reduce weight and control blood glucose without receiving the second-stage LRYGB. As an independent operation, LSG is characterized by relatively simple operation, ability to retain the continuity of digestive tract, small trauma ad a low incidence rate of complications^7^-^9^. Buchwald et al^10^ pointed out that the complete response rate of LRYGB is higher than that of LSG in the treatment of obesity complicated with T2DM, but the opposite conclusion was made by Nocca et al^11^. In this study, the clinical efficacy and safety of LSG and LRYGB in the treatment of obesity patients with T2DM were retrospectively analyzed, so as to provide a strong basis for selecting clinical therapeutic regimens for such patients.

## Materials and methods

### General data

The study collected clinical data from 132 obesity patients with T2DM who were treated with surgery in our hospital between January 2015 and March 2017. The patients were divided into two groups: LSG group (n=66) and LRYGB group (n=66), based on different operation methods. The patients underwent surgical treatment according to the indications formulated by the Chinese Guidelines on the Surgical Treatment of Obesity and T2DM (2014). The inclusion criteria for surgery were as follows: 1) patients with a duration of T2DM ≤15 years, a certain insulin secretion function retained in the pancreatic islet, and serum fasting C-peptide (FCP) ≥1/2 of the lower limit of normal, 2) active surgery was performed for those with body mass index (BMI) ≥32.5 kg/m^2^, and surgery was considered for those with 27.5 kg/m2 ≤BMI ≤32.5 kg/m^2^ and complicated with at least 2 metabolic syndromes (hypertension, hyperlipidemia, and sleep apnea syndrome), and 3) those with a waist circumferenc ≥90 cm (male) and ≥85 cm (female), aged ≥16 years old and ≤65 years old. The study included 68 males and 64 females with an average age of (53.06±9.44) years old. The baseline characteristics of general conditions in the two groups before treatment are shown in Table 1, and there were no statistically significant differences (P>0.05). This study was approved by the Ethics Committee of our hospital, and all patients selected signed the informed consent in accordance with the Declaration of Helsinki. Inclusion criteria were shown below: Duration of T2DM ≤15 years; A certain insulin secretion function retained in the pancreatic islet; Serum fasting C-peptide (FCP) ≥1/2 of the lower limit of normal; BMI ≥32.5 kg/m^2^ or BMI between 27.5 kg/m^2^ and 32.5 kg/m^2^ with at least 2 metabolic syndromes (hypertension, hyperlipidemia, and sleep apnea syndrome); Waist circumference ≥90 cm (male) and ≥85 cm (female); Age between 16 years old and 65 years old. Exclusion criteria were listed as follows: Contraindication for surgery; Pregnancy or lactation; History of severe cardiovascular or cerebrovascular diseases, malignant tumors, or other serious diseases; Mental disorders or cognitive impairment; History of bariatric surgery or other gastrointestinal surgery The clinical data of 132 obesity patients with T2DM treated with surgery in our hospital from January 2015 to March 2017 were collected. The patients were divided into LSG group (n=66) and LRYGB group (n=66) according to different operation methods. Based on the indications of operation formulated by the Chinese Guidelines on the Surgical Treatment of Obesity and T2DM (2014), the following patients underwent surgical treatment 10: 1) patients with a duration of T2DM ≤15 years, a certain insulin secretion function retained in the pancreas islet, and serum fasting C-peptide (FCP) ≥1/2 of the lower limit of normal, 2) active surgery was performed for those with body mass index (BMI) ≥32.5 kg/m^2^, and surgery was considered for those with 27.5 kg/m2 ≤BMI ≤32.5 kg/m^2^ and complicated with at least 2 metabolic syndromes (hypertension, hyperlipidemia and sleep apnea syndrome), and 3) those with a waist circumference ≥90 cm (male) and ≥85 cm (female), and aged ≥16 years old and ≤65 years old. There were 68 males and 64 females aged (53.06±9.44) years old. The baseline characteristics of general conditions in the two groups before treatment are shown in Table 1, and there were no statistically significant differences (p>0.05). This study was approved by the Ethics Committee of our hospital. All patients selected signed the informed consent in accordance with the Declaration of Helsinki.

**Table 1:** Demographics and general clinical data of all studied patients

Parameters	LSG group n=66	LRYGB group n=66	P-value
Gender (Male/Female)	31/35	37/29	0.384
Age (years)	54.74±9.31	53.37±8.67	0.383
BMI (kg/m^2^)	36.95±4.58	38.09±3.64	0.116
Course of disease (years)	8.7±5.1	9.4±5.5	0.449
FBG (mmol/L)	9.60±1.71	10.11±2.20	0.146
2h BG (mmol/L)	17.21±3.63	17.90±2.72	0.209
Fasting plasma C-peptide	1.14±0.31	1.19±0.40	0.424
HbA1C (%)	8.42±1.57	8.56±1.60	0.613
Systemic disease			
Hypertension	19 (50.0%)	23 (45.9%)	0.575
Coronary heart disease	6 (12.2%)	4 (17.6%)	0.744

Notes: LSG: Laparoscopic sleeve gastreetomy; LRYGB: Laparoscopic Roux-en-Y gastric bypass; BMI: Body Mass Index; PBG: Fasting blood-glucose.

### Therapeutic regimens

LSG: Under general anesthesia, routine laparoscopic operation was performed through 4 holes in the umbilicus, below the xiphoid, and on the left and right upper abdomen. The posterior gastric wall was fully dissociated till the left crural diaphragm upwards and 4 cm above the pylorus downwards. Under the support of the 39F stomach tube during operation, the stomach at the side of greater curvature was serially dissected using a laparoscope stapler along the direction of greater curvature from 4-6 cm above the pylorus to His angle, and the volume of residual tubular stomach was about 100 mL. After complete resection, the greater curvature specimen was removed, and whether there were leakage and bleeding inside the cutting line of gastric cavity was checked. LRYGB: Under general anesthesia, routine laparoscopic operation was performed. During operation, most of the distal stomach was separated at the proximal lesser curvature, and the gastric pouch (about 400 mL) was retained and anastomosed with the Roux arm of distal jejunum at 75-100 cm away from the Trietz ligament (anastomotic stoma diameter: 1.75-2.5 cm), and the proximal jejunum stump was anastomosed with the distal jejunum at 75-150 cm (anastomotic stoma diameter: 0.75-1.25 cm), so that most of the distal stomach, the entire duodenum and the proximal jejunum at 75-150 cm were put aside. Then the mesenteric defect was continuously sutured, and each incision was sutured in turn. During the whole operation, the vagus nerve and its branches were separated to avoid damage.

### Observation indexes

The operation time, postoperative hospital stay, total hospitalization expenses, incidence rate of postoperative complications, and preoperative and postoperative use of hypoglycemic drugs were compared between the two groups. The fasting plasma glucose (FPG), fasting insulin (FINS), FCP, glycated hemoglobin A1c (HbA1c), homeostasis model assessment of insulin resistance (HOMA-IR) and HOMA-β were recorded before operation and at 1, 6, 12, 24 and 36 months after operation. FINS was detected using enzyme immunoche-miluminescence method, and HbA1c was detected using latex agglutination method. The insulin resistance index HOMA-IR and the insulin release index HOMA-β were calculated as follows: HOMA-IR = (FPG×FINS)/22.5, HOMA-β = 20×FINS/(FPG - 3.5). The operation effect on T2DM was evaluated. Ineffectiveness: FPG and HbAlc had no obvious improvement compared with those before operation, and the types and doses of hypoglycemic drugs were not significantly reduced compared with those before operation. Improvement: The types or doses of hypoglycemic drugs were significantly reduced compared with those before operation, or FPG could be controlled only through lifestyle intervention after operation, 6.5% ≤post-operative HbA1c <7.5%, postoperative FPG = 5.6-6.9 mmol/L, and 2-hour postprandial blood glucose (2hPBG) = 7.8-11.0 mmol/L for more than 1 year. Complete response: FPG could be controlled only through lifestyle intervention without taking hypoglycemic drugs after operation, HbA1c <6.5%, FPG <5.6 mmol/L, and 2hPBG <7.8 mmol/L for more than 1 year 12. The health-related quality of life of patients after treatment was evaluated using the short-from 36 (SF-36) questionnaire, including 8 dimensions (physical function, role physical, bodily pain, general health, vitality, social function, role emotional and mental health). The lower the score is, the poorer the quality of life in this dimension will be.

### Statistical methods

SPSS 22.0 software was used for statistical analysis. Measurement data were expressed as mean ± standard deviation (*χ̅*±s), and t test was performed for intergroup comparison. Enumeration data were expressed as rate (%), and χ^2^ test was performed for intergroup comparison. P<0.05 suggested the statistically significant difference.

## Results

### Comparison of operation conditions between the two groups

All patients underwent laparoscopic surgery, and no one was switched to laparotomy. Compared with LRYGB group, LSG group had significantly decreased operation time [(108.4±23.1) min vs. (133.8±29.1) min, P<0.001], significantly reduced intraoperative blood loss [(27.5±19.8) mL vs. (34.6±13.3) mL, P=0.017], and significantly shortened postoperative off-bed time [(1.7±0.5) d vs. (2.5±0.7) d, P<0.001]. The postoperative anal exhaust time, postoperative liquid diet time and postoperative hospital stay were all obviously shorter in LSG group than those in LRYGB group, but the differences were not statistically significant (P>0.05). The numeric rating scale (NRS) score was evidently lower in LSG group than that in LRYGB group at 1 d after operation [(3.8±0.5) points vs. (4.8±0.8) points, P<0.001], while it had no statistically significant difference at 3 d after operation (P In LSG group and LRYGB group, there were 4 cases and 6 cases of fever, and 2 cases and 4 cases of abdominal distension, respectively, which were improved after medical conservative treatment. Mild reflux occurred in 1 case and 2 cases, respectively, in the two groups, and it was improved after treatment with acid-suppressing drugs and gastric mucosal protective agents. In LRYGB group, incision infection was found in 2 cases, and 1 case had abdominal hemorrhage complicated with subphrenic infection after operation, confirmed as short gastric artery branch hemorrhage later, which was relieved through hemostasis, acid-suppressing therapy, antibiotic therapy and fasting. None of patients suffered from anastomotic stenosis, obstruction, ulcer, bleeding, gastrointestinal fistula and deep vein thrombosis after operation (Table 2). Overall, Laparoscopic sleeve gastrectomy (LSG) had advantages over laparoscopic Roux-en-Y gastric bypass (LRYGB) in terms of shorter operation time, less intraoperative blood loss, and shorter postoperative off-bed time. However, LRYGB had better outcomes in terms of physical function, general health, vitality, role emotional, and mental health at 12 months after surgery. Both surgeries had similar rates of postoperative complications.

**Table 2:** Comparison of surgery parameters and postoperative complications of patients in the two studied groups

Parameters	LSG group n=66	LRYGB group n=66	P-value
Operation time (min)	108.4±23.1	133.8±29.1	0.001
Blood loss (ml)	27.5±19.8	34.6±13.3	0.017
Postoperative anal exhaust time (d)	1.9±0.6	2.1±0.7	0.080
Postoperative liquid diet time (d)	3.0±0.4	3.1±0.4	0.153
Postoperative off-bed time (d)	1.7±0.5	2.5±0.7	0.001
Postoperative hospital stay time (day)	4.7±1.4	4.9±1.5	0.430
NRS (points)			
1 day postoperative	3.8±0.5	4.8±0.8	0.001
3 days postoperative	1.4±0.4	1.5±0.5	0.207
Postoperative complications	7 (10.6%)	15 (22.7%)	0.135
Fever	4 (6.1%)	6 (9.1%)	
Incision infection	0 (0%)	2 (3.0%)	
Mild reflux	1 (1.5%)	2 (3.0%)	
Abdominal distention	2 (3.0%)	4 (6.1%)	
Abdominal Hemorrhage	0 (0%)	1 (1.5%)	

Notes: LSG: Laparoscopic sleeve gastreetomy; LRYGB: Laparoscopic Roux-en-Y gastric bypass; NRS: Numeric Rating Scales.

### Comparison of operation effect between the two groups

BMI, FPG, FINS, FCP, HbA1c and HOMA-IR all markedly declined in the two groups at each time point after operation compared with those before operation, and the differences were statistically significant (P<0.05). No statistically significant differences were observed in BMI, FPG, FINS, FCP, HbA1c, HOMA-IR and hemoglobin (Hb) between the two groups before operation and at 1, 6, 12, 24 and 36 months after operation (P>0.05). HOMA-β was greatly higher in LSG group than that in LRYGB group at 1, 6 and 12 months after operation (P<0.05), while it had no statistically significant difference between the two groups before operation and at 24 and 36 months after operation (P>0.05) (Table 3).

**Table 3:** Comparison of postoperative therapeutic effect indexes of patients in the two studied groups

	LSG group n=66	LRYGB group n=66	P-value
BMI (kg/m^2^)			
Preoperative	36.95±4.58	38.09±3.64	0.116
1 month postoperative	30.60±3.88	31.32±3.49	0.264
6 months postoperative	26.92±4.56	28.43±5.35	0.083
12 months postoperative	25.77±4.69	26.41±4.32	0.416
24 months postoperative	25.36±4.38	26.05±4.17	0.356
36 months postoperative	24.68±3.83	25.11±3.30	0.491
FBG (mmol/L)			
Preoperative	9.60±1.71	10.11±2.20	0.146
1 month postoperative	7.41±0.83	7.53±0.92	0.433
6 months postoperative	6.58±0.79	6.67±0.74	0.501
12 months postoperative	5.93±0.87	6.04±0.88	0.472
24 months postoperative	5.64±0.79	5.71±0.84	0.623
36 months postoperative	5.14±0.91	5.22±0.89	0.611
FINS (µIU/ml)			
Preoperative	35.80±16.76	37.21±18.09	0.643
1 month postoperative	27.54±11.42	28.93±12.07	0.498
6 months postoperative	22.39±14.31	23.69±13.58	0.593
12 months postoperative	16.89±10.36	17.78±10.72	0.629
24 months postoperative	11.43±7.99	12.51±7.84	0.435
36 months postoperative	9.06±5.88	9.90±5.62	0.403
Fasting plasma C-peptide (ng/ml)			
Preoperative	1.14±0.31	1.19±0.40	0.424
1 month postoperative	0.89±0.30	0.92±0.34	0.592
6 months postoperative	0.91±0.36	0.94±0.37	0.638
12 months postoperative	0.90±0.41	0.92±0.45	0.790
24 months postoperative	0.85±0.44	0.88±0.46	0.702
36 months postoperative	0.84±0.50	0.87±0.47	0.723
HbA1C (%)			
Preoperative	8.42±1.57	8.56±1.60	0.613
1 month postoperative	7.21±0.90	7.34±0.91	0.411
6 months postoperative	6.89±0.95	6.94±1.04	0.774
12 months postoperative	6.67±1.02	6.74±0.97	0.687
24 months postoperative	6.23±0.87	6.33±0.79	0.491
36 months postoperative	5.77±0.76	5.85±0.69	0.528
Homa-IR			
Preoperative	8.98±4.91	10.34±4.45	0.098
1 month postoperative	4.80±4.11	5.15±4.70	0.650
6 months postoperative	4.74±2.93	4.88±2.04	0.751
12 months postoperative	4.66±1.36	4.97±1.69	0.248
24 months postoperative	3.52±1.72	3.76±1.95	0.455
36 months postoperative	2.79±1.86	3.10±1.90	0.345
Homa-β			
Preoperative	176±81	155±74	0.122
1 month postoperative	188±96	149±92	0.019
6 months postoperative	191±99	144±94	0.006
12 months postoperative	207±159	141±90	0.001
24 months postoperative	168±108	139±68	0.067
36 months postoperative	153±135	126±67	0.148
Hb (g/L)			
Preoperative	140.6±17.6	138.8±15.6	0.535
1 month postoperative	138.9±15.5	137.9±15.0	0.707
6 months postoperative	138.3±13.3	137.5±14.8	0.744
12 months postoperative	137.1±11.7	136.4±12.1	0.636
24 months postoperative	136.6±10.5	135.3±12.4	0.517
36 months postoperative	135.5±12.9	134.0±11.6	0.484

Notes: LSG: Laparoscopic sleeve gastreetomy; LRYGB: Laparoscopic Roux-en-Y gastric bypass; BMI: Body Mass Index; FBG: Fasting blood-glucose; FINS: Fasting insulin; Hb: Hemoglobin.

The patients were followed up for 36 months, and the condition of T2DM was improved in the two groups after operation compared with that before operation. There were no statistically significant differences in the complete response rate at 12, 24 and 36 months after operation between LSG group and LRYGB group (68.2% vs. 71.2%, 74.2% vs. 78.8%, 80.3% vs. 87.9%) (P>0.05) (Table 4). Taken above, Both LSG and LRYGB groups showed significant improvement in BMI, FPG, FINS, FCP, HbA1c, and HOMA-IR after surgery, and there were no significant differences in these parameters between the two groups at various time points. LSG group had significantly higher HOMA-β at 1, 6, and 12 months after surgery. The complete response rate of T2DM at 12, 24, and 36 months after surgery showed no significant differences between the two groups.

**Table 4:** Clinical effective rates of the two studied groups

	LSG group n=66	LRYGB group n=66	P-value
12 months postoperative			0.850
Complete response	45 (68.2%)	47 (71.2%)	
Improved	21 (31.8%)	19 (28.8%)	
24 months postoperative			0.682
Complete response	49 (74.2%)	52 (78.8%)	
Improved	17 (25.8%)	14 (21.2%)	
36 months postoperative			0.341
Complete response	53 (80.3%)	58 (87.9%)	
Improved	13 (19.7%)	8 (12.1%)	

Notes: LSG: Laparoscopic sleeve gastreetomy; LRYGB: Laparoscopic Roux-en-Y gastric bypass.

### Postoperative SF-36 scores in the two groups

At 12 months after operation, the scores of physical function, general health, vitality, role emotional and mental health in the SF-36 questionnaire were all remarkably higher in LRYGB group than those in LSG group (P<0.05), while the scores of role physical, bodily pain and social function had no statistically significant differences between the two groups (P>0.05) (Table 5).

**Table 5:** Comparison of postoperative SF-36 life quality scale scores of the studied patients in two different groups

Complications	LSG group n=66	LRYGB group n=66	P-value
Physical function	72.94±16.98	79.60±17.09	0.026
Role physical	46.43±9.64	48.06±8.19	0.297
Bodily pain	66.71±11.39	64.11±12.67	0.217
General health	64.53±15.65	70.14±15.90	0.043
Vitality	58.79±13.27	63.31±12.67	0.047
Social function	72.09±14.89	74.24±13.35	0.384
Role emotional	64.57±10.45	70.63±11.33	0.002
Mental Health	73.39±11.45	80.04±12.63	0.003

Note: LSG: Laparoscopic sleeve gastreetomy; LRYGB: Laparoscopic Roux-en-Y gastric bypass.

## Discussion

T2DM is a chronic disease affecting more than 400 million people in the world. The risk of T2DM in obesity patients rises 13. It was pointed out in a related guideline that surgical intervention is recommended for obesity-related complications, such as DM 14. In foreign literature, it is recommended that surgical treatment be performed for T2DM patients with BMI >35 kg/m^2^ 15. In view of the characteristics of Chinese population, Chinese guidelines suggest that T2DM patients with BMI >32.5 kg/m^2^ undergo active operation 12. 3D laparoscope has high resolution and clear view, which can well reveal the hierarchical structure of tissues at the operative area. Due to its visual depth, surgeons can feel the structure far beyond the ultrasonic knife, electric scalpel or separating forceps at all times, and the spatial positioning becomes clearer, so it is easier to distinguish the correct layer, making the surgical operation more precise, thus improving the surgical safety^16^,^17^. LRYGB and LSG are the two most widely-used surgical methods currently, and their efficacy, especially long-term efficacy, remains controversial. Yska et al^18^ conducted a study for 569 T2DM patients who underwent different bariatric surgeries in the UK, and found that the response rate of DM is higher in patients receiving LRYGB than that in patients receiving LSG. Buchwuld and Oien pointed out that the complete response rate of DM is about 85% after LRYGB and 46% after LSG, and the efficacy of LRYGB is better than that of LSG^19^. Peterli et al^20^ also argued that LRYGB possesses the effects of restricting food intake and reducing nutrient absorption, and the response rate of DM by LRYGB should be higher than that by LSG from the perspective of “foregut hypothesis” and “hindgut hypothesis”. However, it was found in most studies that there is no statistically significant difference in the hypoglycemic effect between the two surgical methods in the early follow-up^21^.

The research results of Schauer et al^22^ showed that the hypoglycemic effect has no statistically significant difference between the two surgical methods at 1 year after operation, but the hypoglycemic effect of LRYGB is better than that of LSG after 3 years. However, Rosenthal and other international experts in LSG proposed based on the data of more than 12,000 patients that LSG has reliable efficacy and can absolutely be used as an independent surgical method for T2DM 23. Moreover, recent studies have confirmed that there is no difference in the long-term efficacy between the two surgical methods. According to the randomized controlled study of Salminen et al^24^, the response rate of DM has no statistically significant difference among T2DM patients treated with LSG and LRYGB at 5 years after operation.

A large number of clinical research results have demonstrated that LSG has definite effects of reducing body weight and controlling blood glucose, and its glucose-controlling effect has no statistically significant difference compared with GB. The clinical efficacy of LSG is usually satisfactory, and in the case of poor control effect on DM or postoperative recurrence, a variety of second-stage operation methods can be used, including ileal interposition, duodenal jejunal bypass, and side-to-side jejunoileal anastomosis. The second-stage GB not only destroys the residual sleeved stomach, and resects the regulator valve for gastric emptying, but also excludes the duodenum, so it is not the optimal solution.

Therefore, it is recommended by some scholars that LSG be used as the first-stage operation, and related intestinal surgery be done additionally if diabetes treatment fails or the disease relapses. In this study, BMI, FPG, FINS, FCP, HbA1c and HOMA-IR were all significantly improved in LRYGB group and LSG group at each time point after operation compared with those before operation, and the response rate of DM had no statistically significant difference between the two groups, indicating that the efficacy of LSG and LRYGB is similar, Moreover, LSG grouhad shorter operation time, less bleeding and quicker postoperative recovery. There was no statistically significant difference in the Hb level between the two groups before operation and at each time point after operation (P>0.05), suggesting that no nutritional anemia occurred due to changes in digestive tract structure or appetite reduction after LRYGB and LSG.

LRYGB and LSG alter the original structure of the digestive tract and reduce the gastric volume. After operation, therefore, some patients suffer from mild gastroesophageal reflux, abdominal distension and other gastrointestinal complications, and some have physical decline, anxiety and mental tension. In this study, the scores of physical function, general health, vitality, role emotional and mental health in the SF-36 questionnaire were all remarkably higher in LRYGB group than those in LSG group at 12 months after operation (P<0.05). The possible reason is that LRYGB retains the residual gastric volume as far as possible during operation, so that the restriction on food intake in daily life is reduced, preventing the phenomena of “quickly stuffed”, “heartburn and acid reflux” and “physical decline”, and raising the quality of life of patients to a certain extent. LRYGB and LSG had no obvious difference in improving bodily pain in the SF-36 questionnaire (P>0.05). The possible reason is that diabetic patients mainly have visceral autonomic nerve dysfunction, so the bodily pain is not obviously improved after operation. This single-center retrospective study has several limitations that need to be considered when interpreting its results.

Firstly, the sample size of this study was relatively small, which may limit the generalizability of the findings to the wider population. A larger sample size would have increased the statistical power and provided more reliable results. Secondly, the follow-up period in this study was not long enough, and therefore, the long-term outcomes of the intervention are uncertain. Longer follow-up periods are required to assess the durability of the treatment effect and to identify any potential late complications. Thirdly, the follow-up content in this study was not comprehensive enough, and important variables such as comorbidities, adherence to treatment, and patient-reported outcomes were not adequately evaluated. These variables could significantly affect the treatment outcomes and, therefore, their absence may limit the accuracy of the study results. Fourthly, as this was a retrospective study, there may be some degree of selection bias, which can occur when patients are not randomly assigned to treatment groups. Finally, the lack of a control group in this study is also a significant limitation. Without a control group, it is difficult to ascertain whether the observed treatment effect is attributable to the intervention or other factors such as natural disease progression, placebo effect, or other concurrent treatments. Therefore, to confirm the findings of this study, larger prospective multicenter randomized controlled studiewith longer follow-up periods and comprehensive assessment of relevant variables are needed.

## Conclusion

In summary, This study found that both laparoscopic sleeve gastrectomy (LSG) and laparoscopic Roux-en-Y gastric bypass (LRYGB) are effective in treating obesity with type 2 diabetes mellitus (T2DM), with similar weight reduction and glucose-lowering effects. LSG has the advantage of being a simpler procedure with smaller trauma and quicker postoperative recovery, while LRYGB is associated with better quality of life for patients. These findings suggest that both procedures can be considered as effective treatment options for obese patients with T2DM, but the choice of procedure should be made based on individual patient characteristics and preferences. Further large-scale prospective studies are needed to confirm these findings.
